# Anaphylaxis after consumption of wasp larvae in Reunion Island: a case report

**DOI:** 10.3389/falgy.2023.1213879

**Published:** 2023-06-16

**Authors:** Adrien Maillot, Camille Mathelin, Gregory Cazanove, Adrien Marteau

**Affiliations:** ^1^ Inserm CIC 1410, University Hospital, Saint Pierre, Réunion Island, France; ^2^ Indian Ocean Toxicovigilance Department, University Hospital, Saint-Denis, Réunion Island, France; ^3^ Department of Emergency, University Hospital, Saint Pierre, Réunion Island, France; ^4^ Museum of Natural History, Saint Denis, Réunion Island, France

**Keywords:** entomophagy, allergy, *Polistes olivaceus*, Indian ocean, edible insects

## Abstract

The practice of entomophagy is common in Asia, Africa, and South America and is now spreading to Europe and the United States. Entomophagy is not without risk since humans can develop allergic reactions to the ingested insects. Here we describe a case of anaphylaxis after consumption of *Polistes olivaceus* larvae in a 23-y-old man living in Reunion Island, a French overseas department where wasps and other insects are occasionally consumed as part of local traditions. The patient developed diffuse pruritus with facial edema, nausea, and vomiting 15 min after ingesting pan-fried wasp larvae during a dinner with two other people. He was taken to a local care center where he received two oral doses of antihistamines. Shortly after, he presented with shock and hemodynamic, respiratory, and neurological failure. He received a subcutaneous injection of adrenaline and was rapidly transferred to hospital for 12 h of monitoring, after which he was discharged without sequelae. The patient's anaphylactic reaction may have been due only to the allergens contained in the ingested larvae or to cross-allergy. To our knowledge, this is the first reported case of anaphylaxis after consumption of *Polistes olivaceus* larvae. More generally, few cases of allergic reaction to ingested insects have been described in the literature.

## 1. Introduction

Entomophagy, the human consumption of insects as food, is a common practice in Asia, Africa, and South America, with an estimated 2 billion people incorporating insects in their diet worldwide (1). Though still marginal, the practice in now spreading to Europe and the United States (2, 3). As insects contain good fats, vitamins, and high levels of protein, calcium, iron, and zinc, they provide a nutritious alternative to conventional meat (1, 4). The *Edible Insects* report published by the Food and Agriculture Organization recommends developing insect farming on a large scale to achieve global food security (1).

**Figure 1:**
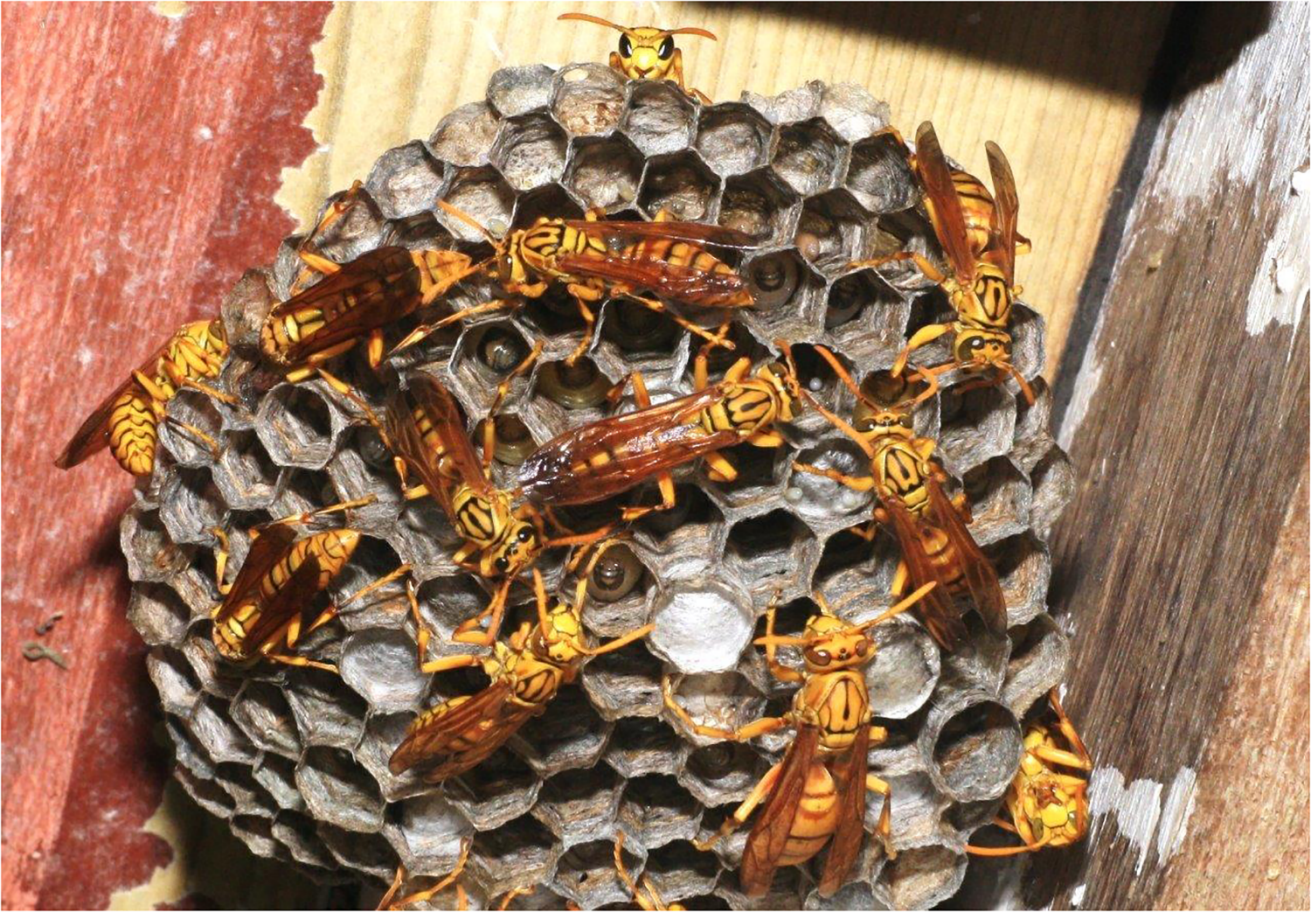
*Polistes olivaceus* taking care of their larva. Reunion Island. Copyright Jean-Maurice Tamon.

**Figure 2:**
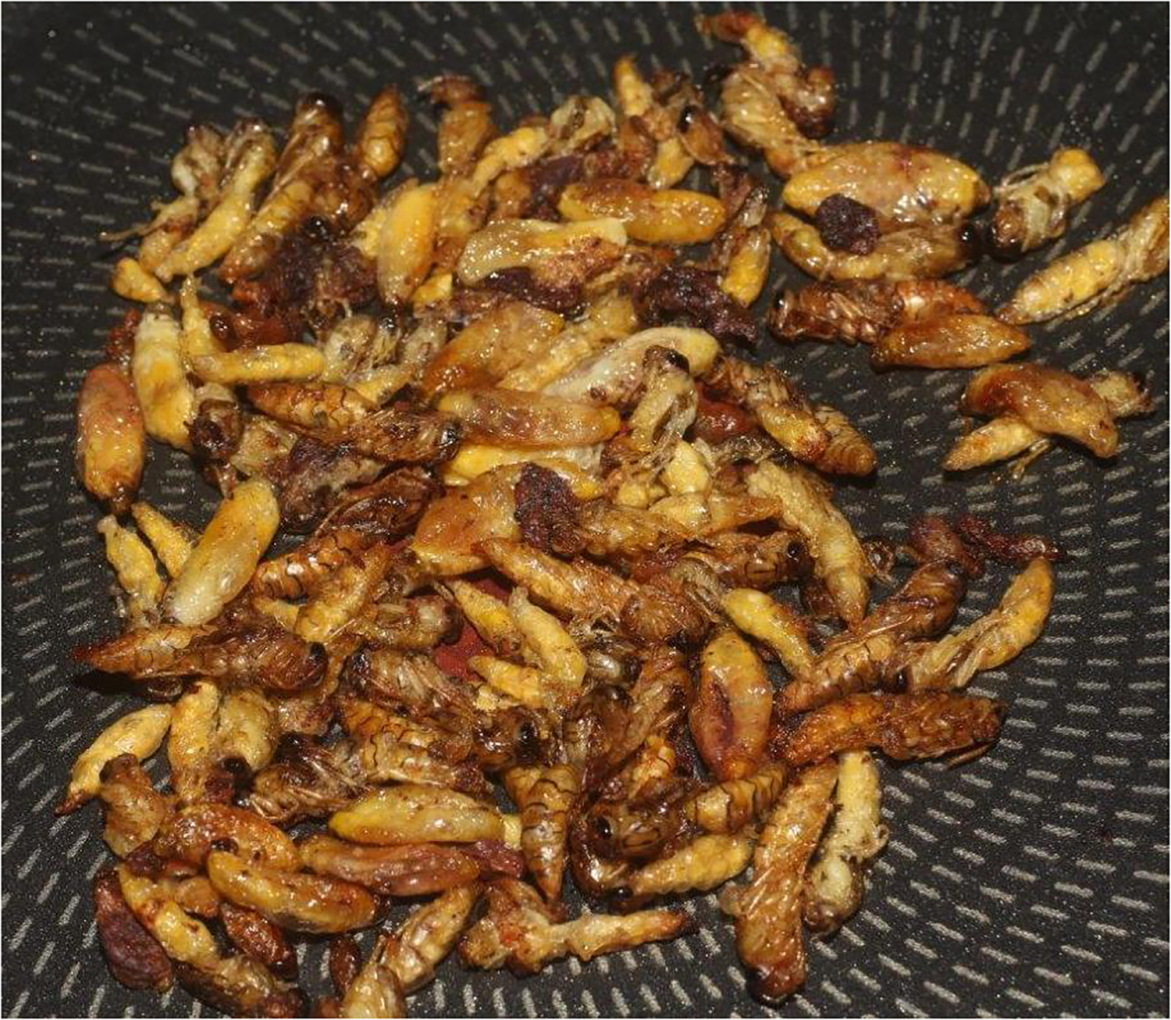
Fried-pan *Polistes olivaceus* larvae and pupae. This photo is only illustrative; it does not directly concern our patient who consumed only larvae. Reunion Island. Copyright Jean-Maurice Tamon.

More than 2,000 species of insects are known to be consumable by humans (5). Hymenoptera (wasps, bees, and ants) account for 15% of edible insect species, followed by coleopteran (beetles), lepidoptera (butterflies), orthopteran (grasshoppers), etc. In the French overseas department of Reunion Island, wasp larvae are occasionally consumed as part of local traditions—unlike the situation in many parts of Africa, where they constitute an alternative source of food (6). Reunionese extract larvae from wasp nests (Figure 1) before the winter season and sometimes sell them at a high price on the local market. Typically, the wasp larvae are cooked with sunflower oil in a pan over high heat for a few minutes, and are then seasoned with salt and pepper before serving (Figure 2).

Here we describe the case of a 23-y-old man living in Reunion Island who developed anaphylaxis approximately 15 min after ingesting *Polistes olivaceus* larvae. Few cases of allergic reaction to ingested insects have been described in the literature, and to our knowledge this is the first reported case of anaphylaxis after consumption of *Polistes olivaceus* larvae.

## 2. Case description

A 23-y-old man with a history of asthma treated with budesonide/formoterol was admitted to the emergency room of Reunion Island University Hospital for symptoms of anaphylaxis. During a meal with two other people, the patient had developed pruritus with facial edema, nausea, and vomiting approximately 15 min after ingesting 150 g of wasp larvae pan-fried for 10 min. He was immediately taken to a local care center, where he received 2 oral doses of dexchlorpheniramine. His health condition deteriorated rapidly: he presented with sweating, drowsiness with a Glasgow Coma Scale score of 13, polypnea without sibilants or uvula edema, and an arterial blood pressure of 60/40 mm Hg (heart rate was non-measurable). A general practitioner gave him 0.8 mg of adrenaline subcutaneously.

When emergency services arrived 20 min later, the patient's health condition had stabilized: his arterial blood pressure had risen to 145/75 mm Hg, his heart rate was 85 beats·min^−1^, his consciousness was restored (Glasgow Coma Scale score of 15), and the electrocardiogram was normal. The patient was given 80 mg of prednisolone orally and was transferred to Reunion Island University Hospital for monitoring.

The blood test performed on admission showed an elevated white blood cell count (14.45 G·L^−1^; normal range 4.00–10.00 G·L^−1^), fibrinogen levels below normal (1.87 g·L^−1^; normal range 2.00–4.00 g·L^−1^), and slightly elevated troponin levels (35 ng·L^−1^; normal range 0–34 ng·L^−1^). The rest of the blood test was normal. A serum tryptase assay performed 3 h after the allergic episode showed significantly elevated tryptase levels (30.5 µg·L^−1^; normal range < 11.4 µg·L^−1^). During the clinical interview, the patient reported having developed a discrete rash with no other clinical manifestations after eating *Polistes olivaceus* larvae for the first time a year earlier.

The patient was discharged without sequelae 12 h after admission. He was prescribed antihistamine H1 and corticosteroids for 3 days as well as an adrenaline injector (with instructions on when and how to use it). Nine months after the anaphylactic episode, he saw an allergist who prescribed him an allergy test and a lung function test: the allergy test was never performed and the lung function test came back normal.

## 3. Discussion

Food allergies to insects have rarely been described in the literature. In Asia, where entomophagy is common, the estimated prevalence of anaphylaxis due to insect consumption ranges from 0.3 to 19.4% (7). This wide variation in reported prevalence rates may be explained by the non- standardization of methodologies and the underreporting of allergic episodes caused by non-identified insect species. To our knowledge, ours is the first reported case of anaphylaxis after consumption of *Polistes olivaceus* larvae.

Our patient developed severe anaphylaxis after eating pan-fried *Polistes olivaceus* larvae. Several of the criteria recommended by consensus for the clinical diagnosis of anaphylaxis (4–5) were present: acute onset (15 min) of diffuse pruritus with persistent nausea and vomiting followed by dyspnea and collapse with systolic blood pressure below 90 mm Hg. It is now recognized that cofactors play a fundamental role in the onset of anaphylaxis, with the study by Wölbing et al. reporting cofactors in about 30% of anaphylactic reactions (8). In our patient, asthma was a clear cofactor in the onset of anaphylaxis.

Although the diagnosis of anaphylaxis is usually established clinically, it can be reinforced using different biomarkers (9). The measurement of serum total mast cell tryptase is the current gold standard laboratory test for food allergies, as elevated tryptase levels can indicate mast cell activation, which is responsible for vascular vasodilation and bronchial hyperresponsiveness (10, 11). In our patient, the presence of elevated total serum mast cell tryptase levels (>10.4 ng·L^−1^) reinforced the diagnosis of anaphylaxis. However, the absence of elevated tryptase levels or other biomarkers does not indicate a lack of allergic reaction. In the study by Sala-Cuill et al., tryptase levels were elevated in only about 60% of adults with a clinically confirmed diagnosis of anaphylaxis (12). The low precision of biomarkers may be explained by the fact that the pathophysiology of anaphylaxis involves the activation of multiple pathways, cell types, and mediators (13). According to the studies that have already been conducted, the variation of the cut-off value can affect the diagnostic performance of the test (14). The difficulty of conducting a clinical study in an emergency room setting, taking into account all the conditions required to perform the measurement of serum total mast cell tryptase, does not allow an optimal cut-off to be set. Although serum tryptase is the most studied biomarker in anaphylaxis, it is still far from being the ideal biomarker and continues to be debated in the scientific community (15).

Adrenaline is the first-line treatment for anaphylaxis (9). Our patient responded well to subcutaneous adrenaline injection, despite the fact that intramuscular injection in the thigh is recommended due to better absorption (16). The patient's favorable response is a reminder of the fact that the medicines needed to treat anaphylaxis must be available at all times in all health care facilities. Corticosteroids are often used for the emergency treatment of anaphylaxis, even though they are not recommended because of unproven efficacy. They are, however, indicated to prevent biphasic anaphylactic reactions (17, 18). Antihistamines can be effective against cutaneous-mucosal symptoms, but not against anaphylaxis (19). The pruritus observed in our patient was reduced following the administration of dexchlorpheniramine.

*Polistes* is a cosmopolitan genus with more than 200 species, most of which are found in tropical or subtropical regions (20). The adult of *Polistes olivaceus* (De Geer, 1773) is easily recognizable with its ochre-yellow body, legs, and antennae and its pattern of narrow brown stripes. Since wasp larvae are devoid of venom gland, the components usually found in a wasp sting envenomation cannot explain the allergic reaction observed in our patient. Our main hypothesis is that the onset of anaphylaxis was caused by an allergen present in the larvae, which is supported by the fact that the patient was stung by a *Polistes olivaceus* wasp without developing an allergic reaction one year after the anaphylactic episode. Primary sensitization was also likely since in our patient reported having developed a discrete rash after eating wasp larvae for the first time a year earlier.

Some of the insect allergens responsible for food allergies have been identified. Among these, tropomyosin and arginine kinase are pan-allergens that can give rise to cross-reactivity with homologous proteins in crustaceans and dust mites (21–23). The patient has not reported a lifetime allergic event and therefore has never consulted an allergist before this event. However, it is possible to hypothesize that a primary sensitization to an allergen may be the cause of a secondary food allergy (24). The onset of anaphylaxis in our patient may have been caused by exposure to one or both of these pan-allergens.

More data are needed to understand the mechanisms underlying food allergy to insects.

This is the first reported case of anaphylaxis after consumption of *Polistes olivaceus* larvae. Given the likely development of entomophagy in the years to come, more data are needed to raise awareness about the risk of allergic reaction to ingested insects.

## Data Availability

The original contributions presented in the study are included in the article, further inquiries can be directed to the corresponding author.
